# *Taxus baccata* L. Under Changing Climate Conditions in the Steppe Zone of the East European Plain

**DOI:** 10.3390/plants14131970

**Published:** 2025-06-27

**Authors:** Vladimir Kornienko, Alyona Shkirenko, Valeriya Reuckaya, Besarion Meskhi, Dmitry Dzhedirov, Anastasiya Olshevskaya, Mary Odabashyan, Victoria Shevchenko, Dzhuletta Mangasarian, Natalia Kulikova

**Affiliations:** 1 Scientific Research Laboratory for Monitoring and Forecasting of Donbass Ecosystems, Donetsk State University, 24 Universitetskaya St., 83001 Donetsk, Russia; alyona.shkirenko@mail.ru (A.S.); reutskaya_lerochka@mail.ru (V.R.); 2 Agribusiness Faculty, Don State Technical University, 344000 Rostov-on-Don, Russia; spu-02@donstu.ru (B.M.); ddjedirov@donstu.ru (D.D.); olshevskaya.av@gs.donstu.ru (A.O.); modabashyan@donstu.ru (M.O.); vshevchenko@donstu.ru (V.S.); juliasarkisyan16@yandex.ru (D.M.); nakulikova@donstu.ru (N.K.)

**Keywords:** English yew, climate change, cyclic freezing, dendroecology, steppe zone, mechanical stability of trees, green wood biomechanics, modulus of elasticity, *Taxus baccata*

## Abstract

The aim of the work is to analyze the survival strategy of *Taxus baccata* L., one of the promising plants for landscaping and the creation of woodlands, in the changing ecological conditions of the steppe zone of the Donetsk ridge. In order to achieve this goal, we used biomechanics methods, which help to understand the relationship between the physical and mechanical properties of living tissues and the overall stability of trees during interactions with environmental factors such as temperature, snow and ice storms, cyclic freeze–thaw processes, wind loads, and others. The work was based both on experimental studies on the estimation of the tissue elasticity modulus in response to temperature changes, the mechanical stability of plants, the field collection of materials, and studies on the modeling of forest stand conditions of English yew. As a result of the conducted experiments, it was established for the first time that at the absolute wood moisture content of 77 ± 5.1%, the density of wood tissues in the conditions of Donetsk is 907 ± 43 kg m^−3^. The modulus of elasticity of living tissues depending on the temperature factor varied in the following range: 8.8 ± 0.31 GN m^−2^ (T = 288 K), 11.5 ± 0.55 GN m^−2^ (T = 255 K) and 6.9 ± 0.47 GN m^−2^ (t = 308 K). It was revealed that during the local thawing of skeletal branches and tables, the mechanical resistance of *T. baccata* is reduced by 20–22% and this critically affects the overall plant resistance. It was established for the first time that *T. baccata* in the conditions of the steppe zone has an adaptive strategy of preserving the integrity of the organism under the action of environmental factors with limited loads. The secret lies in the formation of the shape memory effect, under the influence of critical loads. The plant, thus, chooses not migration, not death, but adaptation to changes in environmental conditions, which can become a serious factor in the use of *T. baccata* in the landscaping of urban areas and the creation of artificial forests.

## 1. Introduction

The influence of natural and climatic factors on the mechanical stability of woody plants growing in conditions of anthropogenic pollution has attracted increasing interest over the past 30 years [1,2,3,4,5,6,7,8,9,10,11]. The principles of biomechanics are used to calculate critical loads on skeletal branches or tree trunks [1,6,7,8,9,12,13,14,15,16]. The mechanical stability of plants depends on many factors [2,3,4,6,7,8,9,10,11,12,13,15,16]: load direction, the physical and mechanical properties of the wood (E—modulus of elasticity, MOR—modulus of rupture, ρ), its moisture content (MC, %), plant age, growing conditions and location. The biomechanical properties of wood are species-specific [17,18] and even vary depending on varieties, forms and hybrids.

There are many scientific publications on industrial wood and lumber in the literature, e.g., [19,20,21,22,23,24]. It is not recommended to apply these values to living trees growing in urban conditions, since large errors in calculating mechanical resistance are possible due to the sensitivity of physical and mechanical properties to growing conditions and environmental factors [6,7,9].

The ability of woody plants to resist natural and climatic factors (wind loads, snow and ice rain, etc.) is regulated by their linear dimensions—the length and diameter of the trunk/branches, as well as the direction of the applied load. The length of the trunks and branches affects the bending and torsional moments caused by the loads. Basically, the bending rigidity of the trunk or branch is transferred to the outer annual rings, although they represent only a small fraction of the diameter [25,26]. Allometry plays an important role in studying the relationship between the length and diameter of the trunk/branches [27,28]. It is believed that the constantly changing dimensions of the crown parts of the plant make a greater contribution to the variation in the mechanical stability of the tree than the mechanical properties [29]. In conditions of anthropogenic pollution, under the influence of natural and climatic factors, the importance of the biomechanics of woody plant tissues on the variation in the crown shape remains a poorly studied topic.

Natural factors include wind, precipitation, temperature, air humidity, etc., while anthropogenic factors include soil and air pollution, as well as the vibration and acoustic pollution of the environment. These factors have a particularly acute effect on the ecosystems of Donetsk, as a large and model industrial city [30,31,32,33]. In response to the action of these factors, the physical and mechanical characteristics of woody plants undergo changes. They also change in response to damage by parasites or herbivorous animals [34,35]. Thus, it can be assumed that the viability of woody plants that grow in conditions of anthropogenic pollution will largely depend on the physical and mechanical properties of tissues, which are under the strict action of selection [36].

Mechanical properties of woody plants largely determine their ecological properties, for example, their propagation of vibration signals of animals that are topically associated with them [37,38,39,40,41]; the vibration parameters of trees [42], which affect the interaction of trees with the soil; the illumination under the tree canopy, the value of which affects the formation of the ecological structure of the phytocenosis [9]; and the ability of a tree to support its own weight and withstand external loads, such as the action of wind and precipitation, especially in the form of snow and ice [2,3,4,6,7,8,9,10,15]. In the conditions of a changing climate in the region, most of the questions regarding the mechanical resistance of woody plants to the action of environmental factors, in urban conditions, remain relevant today. There is also an urgent issue of studying and selecting plants (including new species) for urban greening and the restoration of dendrocenoses, which must adapt to new climatic conditions (warm winters, sharp temperature changes within a season, increased freeze/thaw cycles, increased frequency of ice rains and snowstorms). Even those plants that were successfully introduced earlier now require revision in the new conditions.

One of the species that requires close study and a new look in the conditions of Donetsk ridge is *Taxus baccata* L. This species is known to be spread across the north of Norway and Sweden, the east of Estonia, in Poland, the Caspian Sea and Turkey, the south in Greece (including the islands of Euboea, Thasos and Samothraki), the north of Spain, Portugal and Algeria. Large populations are found in Hungary, Slovakia, Ukraine, Crimea, Romania and the Caucasus Mountains. English yew (*T. baccata* L., Taxaceae) is a Tertiary relict locally endangered within parts of its large geographic range [43,44,45,46]. The species is listed in the Red Data Book of the Russian Federation and is assigned to the second category (vulnerable species). Yew populations are characterized by steadily decreasing numbers, which, if further forecasts are made, may soon become endangered [47,48]. At the same time, the state of yew research cannot be recognized as satisfactory. Studies of this species in natural conditions are known, e.g., [48], but there are no data on the influence of environmental factors on the condition of yew plantations in urban conditions (not typical for its distribution), which determines the relevance of the work.

In connection with the above, the main goal of the work is to analyze the survival strategy of *T. baccata*, one of the promising plants for landscaping and the creation of woodlands, in the changing ecological conditions of the steppe zone of the Donetsk ridge.

## 2. Materials and Methods

### 2.1. Object of Study

We chose *T. baccata* as the object of our study. It is not a common species in Donbass, it is found mainly in private collections, in the central part of the city it grows as bonsai, free-growing plants are represented by a small clump in the arboretum of the Donetsk Botanical Garden and one plant is located near the administrative building.

For the research, the samples were divided as follows:

1. A clump of common yew in the steppe zone of the arboretum of the botanical garden was selected. The age of the plant is 48–50 years (according to archival data and cuts from the xylotheque of the scientific laboratory for monitoring and forecasting the ecosystems of Donbass), and it grows without care and artificial watering, in conditions of relative control.
2. Common yew grown by cuttings from mother plants from a clump of the botanical garden. Selected in 2011 and planted as a seedling in 2013. Age 15 years. It is in a private collection, and grows under relative control conditions.

During the experimental part of the work, we divided the studies into two series:

1. The studies were conducted on yew plants growing without the influence of the human factor and only observing the behavior of clumps in natural conditions.
2. A study of wood memory under the constant influence of an observer was conducted. When loading skeletal branches with snow cover, we artificially removed part of the load to prevent the action of the critical mass on the organs of *T. baccata*, thereby disrupting the program for developing the shape memory effect in the body. Depending on the date of the study (meaning, for example, 2017, 2018, 2019, 2022, 2023 years with significant snowstorms and precipitation), the number of load/unload cycles ranged from 2 to 3 to 5 per field observation per day.

The studies were conducted from 2014 to 2025 on clumps of yew (*T. baccata*) in the arboretum of the Donetsk Botanical Garden (10 specimens), single specimens along highways (4 specimens), park areas (6 specimens), as well as growing in private collections (1 model tree).

### 2.2. Dendrological Research

The viability assessment was carried out according to the integral scale of Alekseev with additions on the state of damage to the trunk and crown of the tree [49]: 1 point—healthy plant, 2—weakened, 3—very weakened, 4—dying, 5—deadwood. The results of the visual inspection of the studied woody plants were recorded using a Nikon Coolpix S2600 camera; further office processing and analysis of the images were carried out in the AxioVision Rel. 4.8 program, taking into account the reference measure. The diameter of the trunk and skeletal branches was measured with a caliper.

### 2.3. Conducting Biochemical Studies

The longitudinal modulus of elasticity (E) of wood tissues was determined by a well-known method based on the magnitude of the bending of a cylinder, a cut branch, horizontally clamped in a vice, in response to the application of force at its free end according to Formula (1):

(1) $$E=\frac{64Cl^{3}}{3{\pi d}^{4}},$$

where *C*—cylinder rigidity, *l*—cylinder length, *d*—cylinder diameter.

The rigidity of a cylinder clamped at one end was calculated as follows (2):

(2) $$C=\frac{mg}{x},$$

where m—mass of the applied load, g—acceleration of gravity, x—displacement of the free end of the cylinder.

Calculation of mechanical stability parameters *(*EI, RRB, P_cr_, and m_cr_*)* was carried out according to the following formulas:

Resistance of a tree trunk to bending under dynamic or static loads [12,50]:

Bending resistance = E × I(3)

Relative bending strength (RRB) [12]:

(4) $$RRB=\frac{Er^{2}}{4\rho },$$

where E—modulus of elasticity of green wood fabrics, N × m^2^, I—the second moment of the section, equal to π × r^4^/4; *ρ*—density of living tissues; *r*—radius squared of the trunk or skeletal branches, m.

Critical mass (m_cr_) and maximum permissible load (P_cr_) are parameters that reflect specific values of mass (kg or N), under the action of which the trunk of a tree plant or its skeletal branches begin to deform or break off under the action of wind or gravitational loads [12]:

(5) $$P_{cr}=\frac{EI\pi^{2}}{2l^{2}},$$

(6) $$m_{cr}=\frac{P_{cr}}{g}$$

where EI—bending resistance; l—length of trunk or skeletal branches, m; g—acceleration due to gravity, m/s^2^.

The Microsoft® Excel® LTSC MSO (version 2505, Assembling 16.0.18827.20102) (Microsoft Corporation) was used for statistical data processing. The dependence of the critical mass (m_cr_), bending resistance (EI), and RRB on the *d*/*l* coefficient and trunk diameter for the studied plants was established using a power regression model.

## 3. Results

In Donbass, the yew is an introduced species, which has been actively used in recent years to create highly decorative dendrocenoses. The natural habitat of this species is the Caucasus, Atlantic, Central, Southern, Southeastern Europe, the Mediterranean, Turkey, and Northern Iran.

*T. baccata* is used both in linear plantings along highways in conditions of high anthropogenic load, as a solitary plant, and in formed clumps of many individuals on the territory of the Donetsk Botanical Garden or in a residential area. In the Red Books, the conservation status of this plant is often noted as vulnerable, and its scientific significance is defined as a rare relict species with a disjunctive range.

Representation in the conditions of the city of Donetsk is not high and amounts to 0.15% of the total number of trees studied as of 2025. According to the degree of stability in the conditions of the industrial region, many authors note its good frost resistance and heat tolerance, as well as its high resistance to the action of air toxicants.

### 3.1. Biomechanical Studies

The density of wood tissues in Donetsk City is 907 ± 43 kg m^−3^ at an absolute wood moisture content of 77 ± 5.1%. The elastic modulus of living tissues, depending on temperature, varied within the following ranges: 8.8 ± 0.31 GN m^−2^ (T = 288 K), 11.5 ± 0.55 GN m^−2^ (T = 255 K) and 6.9 ± 0.47 GN m^−2^ (T = 308 K) in the Donetsk Botanical Garden. The differences in biomechanical parameter values are statistically significant (*p* < 0.05).

#### 3.1.1. Effect of Temperature on the Elastic Modulus (E) of Wood Tissues of *T. baccata* In Vitro (Laboratory Studies)

**Figure 1:**
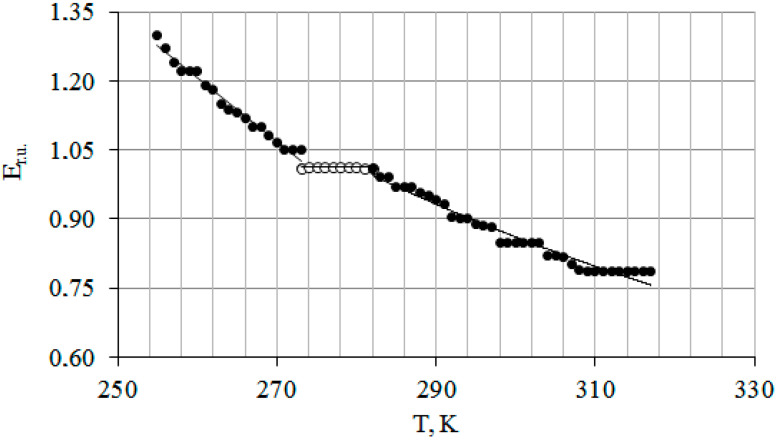
Temperature dependence of the elastic modulus for all studied samples of *T. baccata.* Note: Each value of Er.u. (*T*) is normalized to E = 288 K (E = 8.87 ± 0.65 GN/m^2^). Er.u.—relative modulus of elasticity of living tissue of English yew; T—sample temperature expressed in Kelvin (K). The area with the white circle represents the time period during which ice melting occurs in the plant’s vessels.

The temperature dependence in general for all *T. baccata* samples tends to decrease nonlinearly (closer to the power regression R^2^ = 0.98) with increasing temperature from 255 to 317 K (Figure 1). On the curve Er.u. (*T*), some areas are identified that are out of dependence and have a different angle of inclination. The first section is in the range from 255 to 273 K, the second section is from 273 to 282 K, and the third is from 282 to 317 K. The steepness of the curve Er.u. (*T*) decreases as the rate of temperature increase in the samples decreases. The tilt angles were different in the two sections and this is due to the fact that in our experiment, the temperature of the samples did not remain constant, and the rate of the growth also changed. Thus, it fell as it approached room temperature.

The presence of a temperature gradient between the wood and the surrounding air leads to some abrupt changes in Er.u. Accordingly, such a change in the physical and mechanical properties of wood can be reflected in the rigidity of skeletal branches and the angles at which they depart from the trunk, i.e., in the crown architecture.

The change in the elastic modulus by leaps and bounds during rapid heating is associated with the uneven melting of ice in the volume of the above-ground parts of the plant. In situ, this should lead to an uneven loss of rigidity along the trunk or shoots, and therefore to their deformation at one or more points.

#### 3.1.2. The Influence of Temperature on the Mechanical Stability of Woody Plants *T. baccata* In Situ

The value of the elastic modulus of green wood, and therefore the mechanical resistance of the whole tree, are closely related to the ambient temperature. The most dangerous are not natural transitions from a season with positive temperatures to a season with negative temperatures and vice versa, but temporary sharp jumps in temperature and, in general, climate change within a season (freeze/thaw cycles). We argue that over the past 10 years, the number of such cycles often determines not only the viability, but also the survival of woody plants. There is species-specificity in the effect of the number of cycles, for example, the common peach can withstand no more than two cycles of sharp thawing/freezing (winter season) in the conditions of Donbass, European beech can withstand 2–3 cycles, and common oak can withstand more than five cycles without critical damage. Similar studies on the effect of cyclic processes, but on technical wood, also confirm our field studies [51].

**Figure 2:**
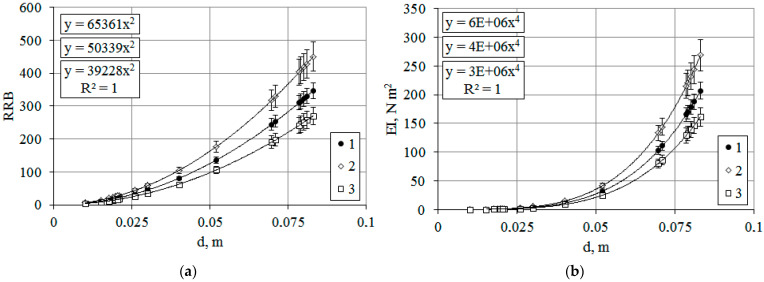
Dependence of the relative bending resistance (**a**) and bending rigidity (**b**) of the trunk of *T. baccata* on its diameter. Note: The simulation was performed in the summer season at *T* = 288 K (1), in the winter season at 255 K (2), and with accelerated defrosting within the season at *T* = 285 K (3). Comment: Y-axis (RRB) data are given in “number·10^7^” format; Y-axis (EI) data are given in “number·10^2^” format.

The following natural and climatic conditions were observed in the experimental area, the effect of which on the *T. baccata* stands will be explained below from the standpoint of biomechanics based on our own mathematical modeling data. After a gradual decrease in temperature to negative values and the transition of plants to a dormant state, a period of prolonged warming began during the winter season; then, over a short period of time, climatic conditions changed dramatically: the air temperature dropped to −20 °C and heavy precipitation fell; wet snow fell for several hours, then it turned into a snowstorm and an ice storm, while gusts of wind up to 15–20 m s^−1^ were recorded. The mechanical stability parameter, the relative bending resistance (RRB), directly depends on the elastic modulus. Since the parameter values change in a quadratic dependence with the thickening of the trunk (Figure 2a), temperature fluctuations will primarily affect the stability of skeletal branches with a minimum trunk diameter, as well as young plants (Figure 2b).

**Figure 3:**
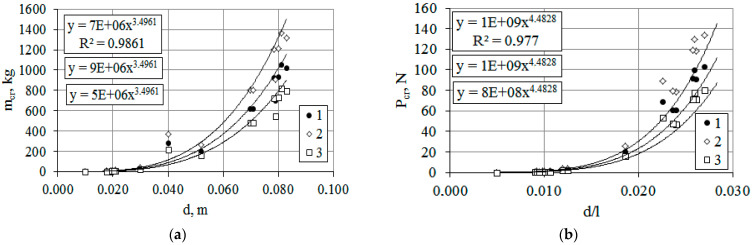
Dependence of critical mass (**a**) on diameter and maximum permissible load (**b**) on the ratio of barrel diameter (d) to its length (l) under environmental factors (ambient temperature). Comment: Y-axis (P_cr_) data are given in “number·10^2^” format.

When assessing the impact of the environmental factor (T, K) on *T. baccata* stands, it should be noted that plants are at risk of irreversible bending during accelerated thawing in winter (Figure 2b, regression Equation (3)). The loss of bending rigidity in this case is 22%. The obtained values reflect the change in the tree’s resistance to mechanical loads, but do not provide an exact value of the critical mass that the plant can withstand or its dimensions at which irreversible deformations or even trunk breakage occur. For this purpose, the calculated values of the critical mass (m_cr_) or the maximum permissible load (P_cr_) on a tree trunk can be used (Figure 3a,b).

The critical mass (m_cr_), as a parameter of mechanical stability, gives exact values, under the action of which irreversible deformations/breakages of the trunk will occur (Figure 3a). For slowly growing *T. baccata* with a small trunk diameter (1–3 cm), the values of the critical load and the maximum permissible load had a minimum indicator, which is especially significant in the winter period for maintaining its own biomass and additional static load (Figure 3a,b regression Equation (2)). The range of the critical mass in young plants (age 7–10 years), due to the slow growth of annual rings, with an allometric ratio *d/l* ≤ 0.011 was 0.7–14 kg (at a temperature of 255 K). In natural conditions of the East European Plain, these values are easily achievable, especially with the combined effect of wind loads [37]. When frozen skeletal branches thaw (which are especially vulnerable at this time due to their green biomass), the critical mass at stress points (due to local heating) decreases by 20–22% at the same values of the trunk diameter (*d*) to its length (*l*) ratio. With an increase in the *d/l* ratio, the critical mass grows in power-law dependence (R^2^ = 0.98) and already at *d/l* > 0.02 exceeds 200 kg.

#### 3.1.3. The Influence of Cyclic Processes on the Mechanical Stability of Woody Plants *T. baccata*

**Figure 4:**
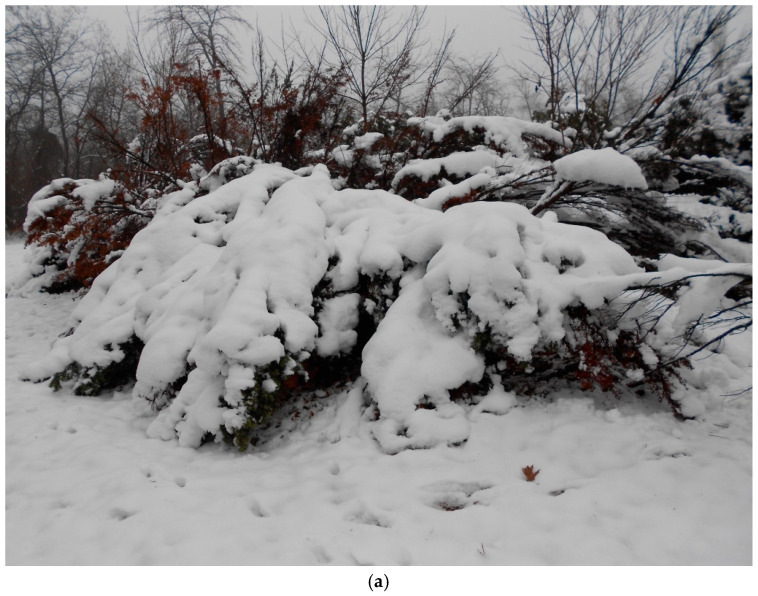

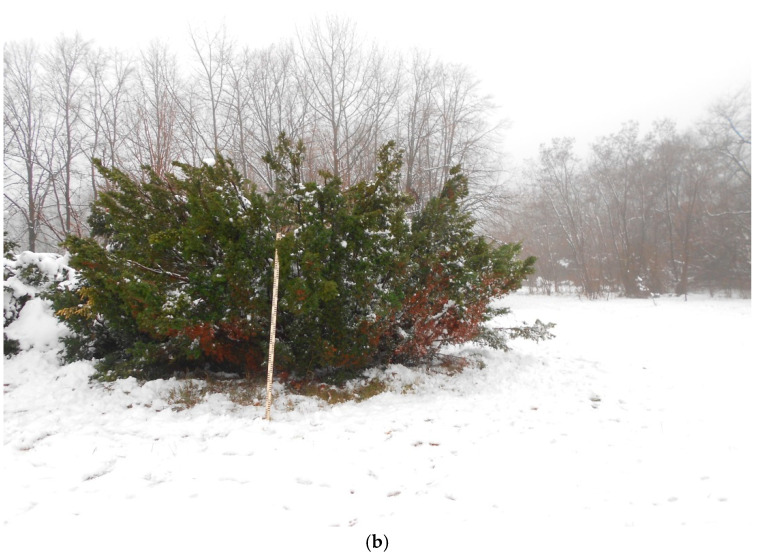
Mechanical resistance of *T. baccata* after the 2017 snowstorm: (**a**) under the load; (**b**) after recovery *(Photo by V. Kornienko, territory of the Arboretum of the Botanic Garden, Donetsk).*

Observation series №1. In situ under conditions of a snow/ice storm and subsequent thawing, the elasticity of skeletal branches changes (for our observations, this is during 2017–2025). In a natural environment (an arboretum of a botanical garden, without human care and influence), a significant volume of snow, during a single strong snowstorm, loaded the skeletal branches of tree stands (first observation on 30 November 2017) and changed the angle of their attachment to the trunk. When the load was removed, the skeletal branches and the crown architecture of the entire tree stand were restored (Figure 4).

**Figure 5:**
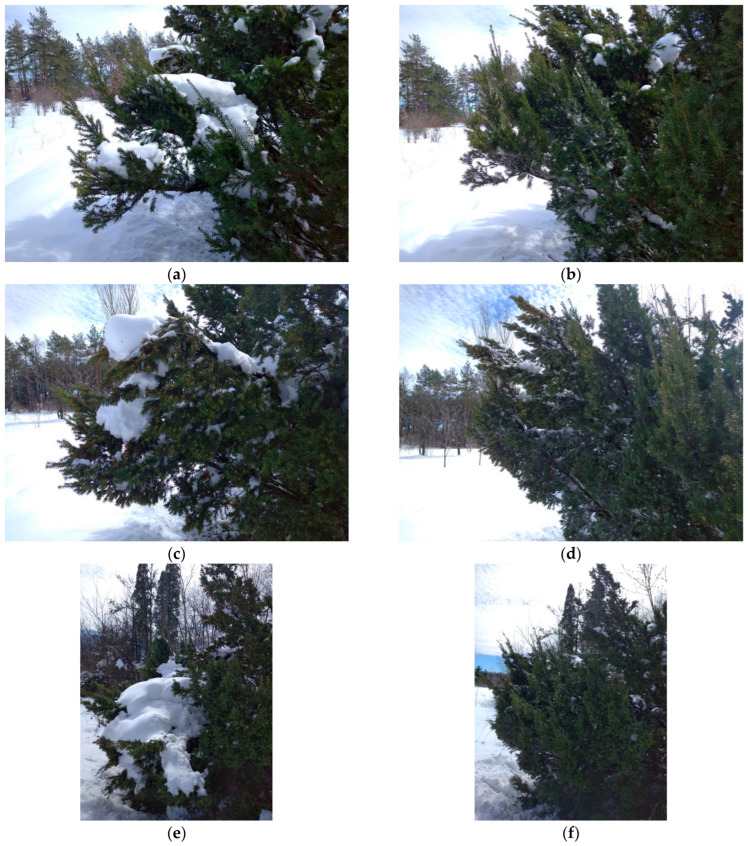
*T. baccata* in the arboretum after a snowstorm, season 2018–2019 (Photo by V. Kornienko, territory of the Arboretum of the Botanic Garden, Donetsk). Notes: (**a**,**c**,**e**)—with load; (**b**,**d**,**f**)—after unloading.

In 2018–2024, repeated snowstorms had a similar effect on *T. baccata* clumps—the ability to operate within the reversible deformation framework and restore its shape remained despite significant precipitation (Figure 5a–f).

**Figure 6:**
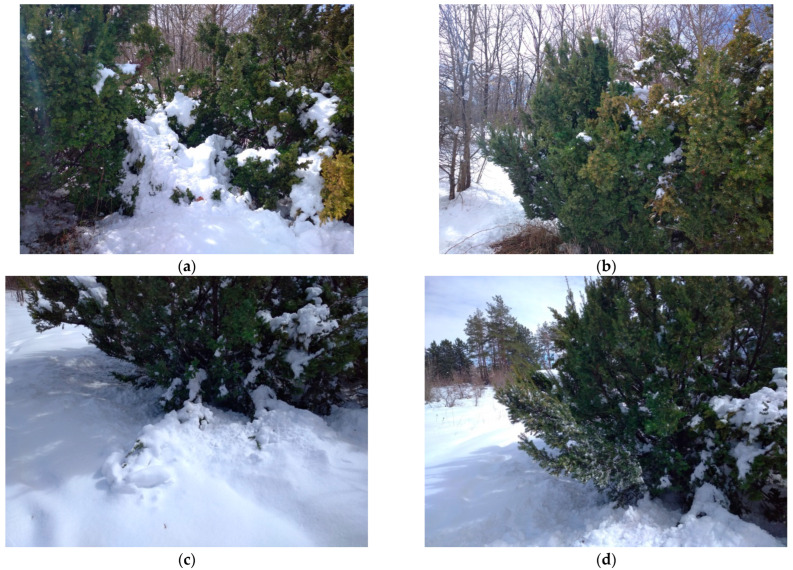
*T. baccata* in the arboretum after a snowstorm, season 2022–2023 (Photo by V. Kornienko, territory of the Arboretum of the Botanic Garden, Donetsk). Notes: (**a**)—with load; (**b**)—after load removal; (**c**)—skeletal branches lie on the soil surface; (**d**)—complete restoration of shape after artificial load removal.

In 2023, during the transition from the season with negative temperatures to the season with positive temperatures, in March after a significant warming, the weather changed abruptly and a significant amount of precipitation fell after a snowstorm (23–24 cm near the *T. baccata* stands). A single load removal freed the crown and the plants restored their vertical position (Figure 6).

**Figure 7:**
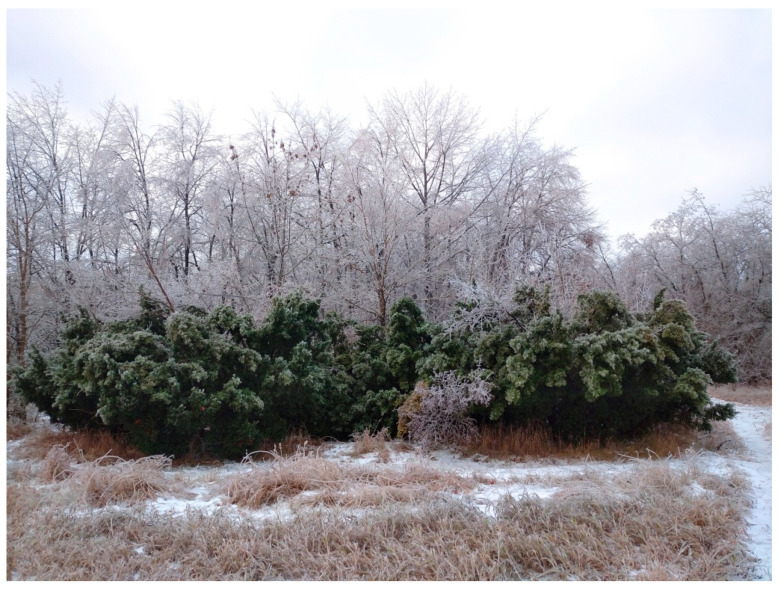
General view of dendrocenosis during freezing rain in 2023 (Photo by V. Kornienko, territory of the Arboretum of the Botanic Garden, Donetsk).

**Figure 8:**
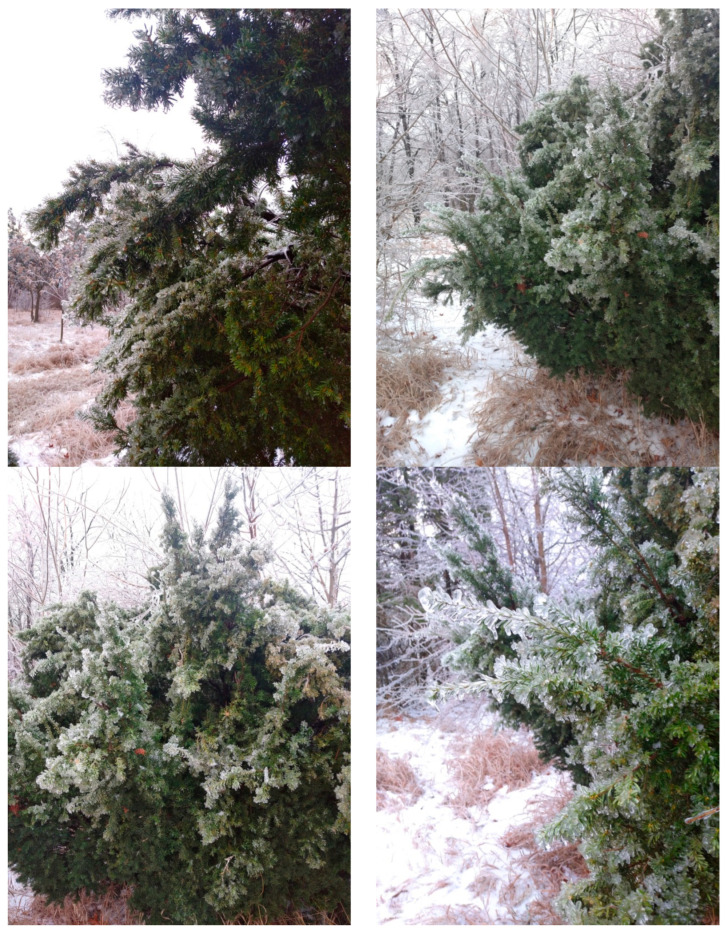
Change in the shape of the crown of the yew tree during the freezing rain of 2023 (Photo by V. Kornienko, territory of the Arboretum of the Botanic Garden, Donetsk).

Freezing rains have become increasingly common in the steppe zone of the East European Plain. In December 2023, we conducted a study of the effects of freezing rain on *T. baccata* clumps growing in the arboretum of the botanical garden and found that due to the lower static load in the form of ice, compared to the adhesion of snow mass, the plants exhibited superior load resistance and remained mechanically stable (Figure 7). The architecture of the damage did not change critically (Figure 8).

**Figure 9:**
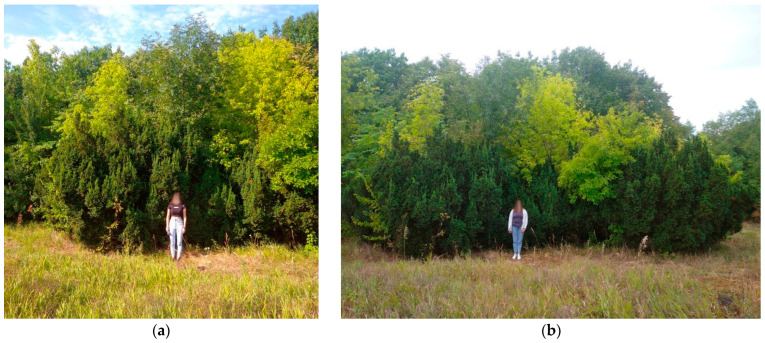
General view of the clump in July (**a**) and September (**b**) 2024 (Photo by V. Kornienko, territory of the Arboretum of the Botanic Garden, Donetsk). Notes: the plants are in excellent condition, growth is vertical.

We noted the functioning of the curtain under a wind load as a single living structure—when one skeletal branch is loaded, the entire system (plant) sways. The natural frequency was 0.6 Hz, and attenuation occurred almost instantly. Locally, the skeletal branches changed their vertical position to a horizontal one; sometimes the top almost tilted by 90 degrees or more. However, after the warming and removal of the load, all plants recovered, and no irreversible deformations or breaks were detected. The 2024/2025 season (without significant snow and ice storms) passed calmly and the plants are in excellent condition (Figure 9).

**Figure 10:**
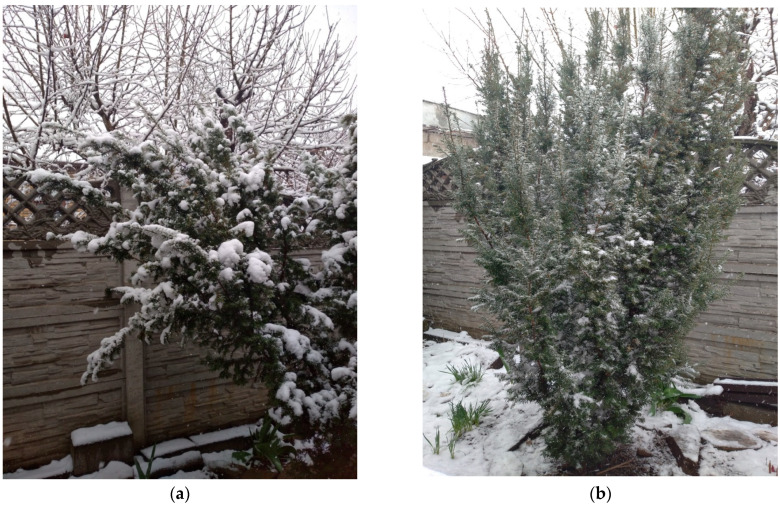
The state of a solitary plant after a single load, in the season of 2022–2023 (Photo by V. Kornienko, territory: private collection of English yew, Donetsk). Notes: (**a**)—with load; (**b**)—after a single artificial load removal.

**Figure 11:**
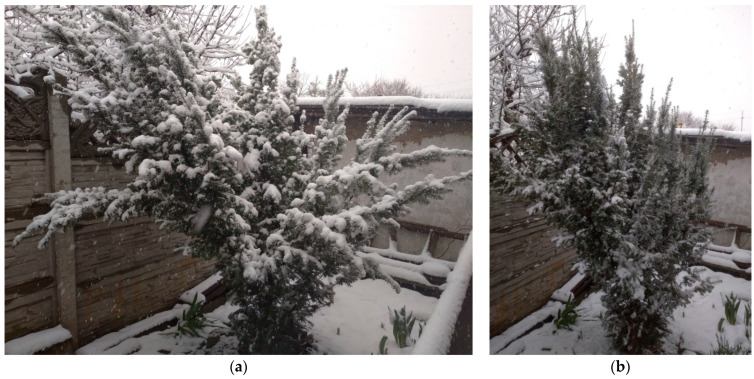
The condition of the solitary plant after two- and three-fold loading, in the season of 2022–2023 (Photo by V. Kornienko, territory: private collection of English yew, Donetsk). Notes: (**a**)—with load; (**b**)—after two- and three-fold artificial removal of load.

**Figure 12:**
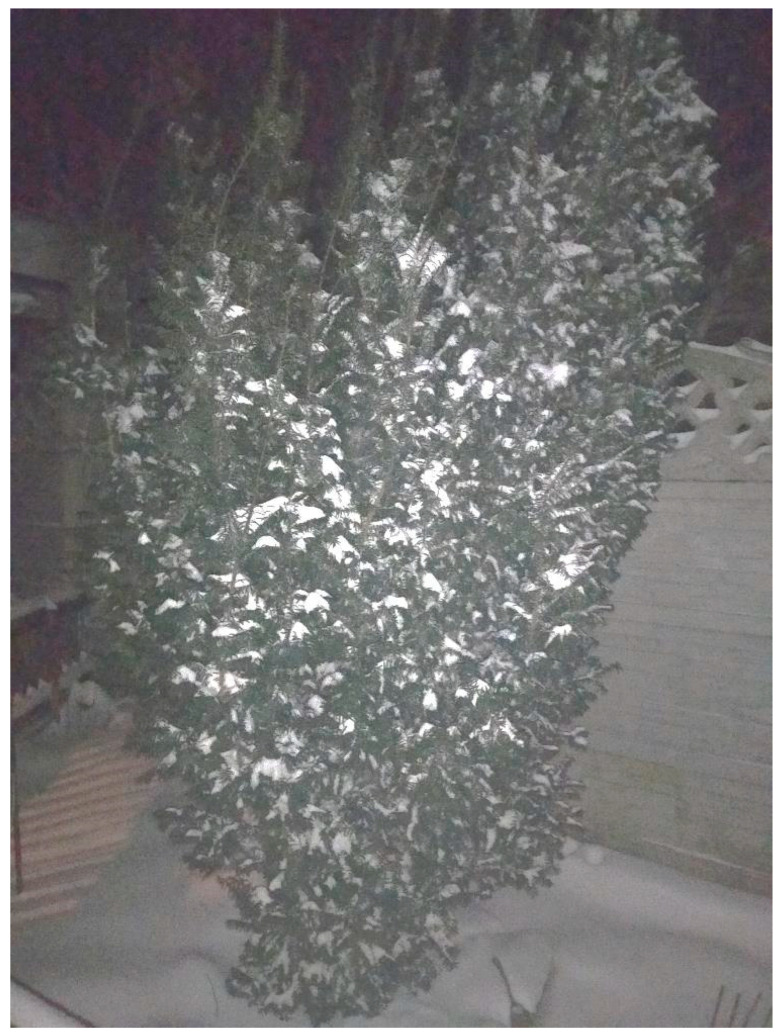
Crown shape preservation during snowfall (from 03:00 h on 08.01 to 23:00 h on 08.01—20 h of precipitation; the crown shape remained unchanged at subzero temperatures) (Photo by V. Kornienko, territory: private collection of English yew, Donetsk). Notes: Nighttime filming was conducted.

Observation series №2. The next series of studies was conducted on self-grown *T. baccata* plants. Observations have been conducted since 2013. The plants were without shelter, additional support and other means of artificially maintaining the vertical position of the organism, which could affect its development and the program for maintaining mechanical stability. In 2023, when precipitation fell (at a positive temperature) in the form of a snowstorm, it was decided to remove the load after each cycle (1 cycle—2 h). With a single load on the skeletal branches, the architecture of the plant crown changed significantly (Figure 10). The angles of deviation from the vertical position had values from 20 to 40 to 116 degrees. When the load was removed, the condition of the crown was restored. After two- and three-fold loading, due to snow sticking to the skeletal branches, complete removal of snow became impossible without damage. Additional mass, already in the form of ice, was preserved (Figure 11). The load was not removed further so as not to damage the plant after the warming the vertical position was restored. In January 2024, with a sharp change in temperature from 276 K to a heavy snowfall with wind gusts of 10–12 m s^−1^ and an air temperature of 262 K the next day, the adhesion of snow as an additional mass practically did not occur. The plant withstood the snowstorm and remained in a vertical position throughout the entire period of the factor’s action (Figure 12).

After the freezing rain in 2023 and 2024, the *T. baccata* retained the strategy of changing the crown architecture after loads above a critical mass. After natural thawing and the complete removal of the load, the crown structure was restored to its original state (vertical normal arrangement of skeletal branches and trunk) after two days.

## 4. Discussion

Paleobotanical and genetic studies show that there is a close relationship between changes in abiotic environmental factors and biological response of plants (physiological, genetic, etc.) [48]. When climatic conditions change, there are several options for plants, either migrating or adapting, otherwise dying [48]. For English yew, adaptation associated with structural and physiological changes turned out to be the preferred survival strategy.

Green wood, in the context of response to climatic factors (static and dynamic loads), can be called a smart material. It is known that under certain loads on the tissues of woody plants, the concept of the deformation memory effect arises [52]. Scientists have also identified the ability of wood to remember the type of applied load, which is important in the context of our ecological studies for *T. baccata*. The shape memory effect (SME) during deformation depends on the load history of the woody plant.

If we take into account that for young plants the critical mass is achieved in the conditions of the steppe zone of Donbass (Figure 3), the ecological consequence of such an impact becomes clear (Figure 4, Figure 5, Figure 6, Figure 7, Figure 8, Figure 10 and Figure 11). It has been found that for English yew trees, when the initial action of a load (e.g., in the form of precipitation) is applied, the plants exhibit the classic response of resisting the applied force as they reach a critical mass acting on the scaffold branches or trunk.

When the critical value is reached, deformation occurs, and the plant tissues try to work in the reversible deformation mode, i.e., with the ability to recover to the original shape. When the same environmental factor (for example, a snowstorm) is applied a second time, the yew is more easily bent, knowing that the organism or even the population will retain its integrity (at most, small frozen skeletal branches that we found under the canopy are sacrificed) by going into a temporary form (curved). In the third cycle and subsequent ones, the entire clump changes the crown architecture even at lower values of the effective real mass (m_cr_), which indicates the action of the shape memory effect on living *T. baccata* plants. This behavior of green wood in response to environmental factors can be explained by quasi-residual “frozen” deformations. After their appearance, changes in intermolecular interactions in tissues occur (primarily this concerns hydrogen bonds) and a significant transformation in the topological structure of wood is observed [53]. When the trunk or skeletal branch returns (i.e., the system functions within the framework of reversible deformation) to its original position (taking into account the absence of microcracks and more significant tissue damage), the disappearance of frozen deformations is observed [53].

When comparing the response of other species growing near English yew stands in the study area, it can be concluded that the behavior of the study object is unique. Thus, under the same climatic conditions and the same conceptual tendency to decrease the elastic modulus with increasing temperature (e.g., locally when some part of the trunk is warmed up), *Juniperus virginiana* L. trees undergo irreversible deformations leading to changes in the crown projection area, thier light permeability and, as a consequence, the temperature and humidity under the canopy [9]. For *Fagus sylvatica* L. trees, irreversible deformations of plant organs, changes in crown architectonics and trunk inclination angle, and in extreme cases, trunk and skeletal branch breakage are typical in the steppe zone under sharp changes in weather conditions within a season (cyclic processes of freezing/thawing) and the uneven warming of woody tissues during thawing [7]. For the two analyzed species, the chosen strategy is either resistance to stress or death. No adaptive response similar to that in English yew was observed. After several snow and ice storms in the region, the trees *J. virginiana*, *F. sylvatica*, *Ulmus pumila* L., *Acer negundo* L., as well as many others, as a consequence of irreversible deformations, lost the integrity of the crown structure, suffered significant damage to plant organs, and significant plant loss. Such ecological consequences are alarming from the point of view of planning decisions for the landscaping of territories, because species are still used which, at this stage of climate change in the region, do not show adaptations and any strategies for survival.

The described results were obtained for the first time in the conditions of the steppe zone of the Donetsk ridge, which is not a typical distribution area of *T. baccata*, and the results are valuable from the point of view of the restoration of forest cover of the territory by species that have shown plasticity in the conditions of the changing climate of the region.

## 5. Conclusions

It was found that at an absolute wood moisture content of 77 ± 5.1%, the density of wood tissues in Donetsk conditions is 907 ± 43 kg m^−3^. The modulus of elasticity of living tissues, depending on the temperature factor, ranged within the following range: 8.8 ± 0.31 GN m^−2^ (T = 288 K), 11.5 ± 0.55 GN m^−2^ (T = 255 K) and 6.9 ± 0.47 GN m^−2^ (T = 308 K).

It was revealed that with local thawing of skeletal branches and tables, the mechanical stability of *T. baccata* decreases by 20–22% and this critically affects the overall stability of plants.

*T. baccata* trees have an adaptive strategy for maintaining the integrity of the organism under the influence of environmental factors with extreme loads. In young plants that experience loads higher than the maximum that is permissible, skeletal branches and even trunks are subject to reversible bending. The slope can reach a critical level, during which the plants bend to the ground and can remain in this state for a long time until the load is removed. This is how the shape memory effect is formed.

When critical loads are applied in several cycles, the flexibility of the organs is higher and the plants change the crown architecture without resistance with each subsequent cycle. After the load is removed, the condition of reversible deformation is met, and the shape of the tree stand is restored. No fallen or irreversibly deformed plants were found.

When the shape memory effect is artificially disrupted by removing the current load, the bending resistance is maintained for a long time.

Practical results of the findings include the following:

1. We recommend introducing *Taxus baccata* L. into the practice of the landscaping of industrial cities of the steppe zone of the Donetsk ridge as an obligatory species that has passed introduction tests for resistance to climate change in the region.
2. We recommend forming both single highly decorative plantings in park zones and forest artificial arrays in order to increase the forest cover of the territory.

## Data Availability

The original contributions presented in the study are included in the article. Further inquiries can be directed to the corresponding author.

## Abbreviations

E	Modulus of elasticity
MC	Moisture content
RRB	Relative bending resistance
SME	Shape memory effect
